# Insights from 25 years of oncogenetics: one person’s perspective

**DOI:** 10.3389/fgene.2023.1180879

**Published:** 2023-05-12

**Authors:** Eitan Friedman

**Affiliations:** The Suzanne Levy Gertner Oncogenetics Unit, The Meirav High Risk Clinic, Sheba Medical Center, Ramat Gan, Israel

**Keywords:** oncogenetic counseling, *BRCA1 BRCA2* genes, high cancer risk, oncogenetic testing, high-risk clinic

## Abstract

In early 1995, I established the oncogenetics service at the Genetics Institute of the Sheba Medical Center in Israel. The purpose of this article is to describe the key points and issues that were raised throughout my personal journey since then: physician and public awareness; ethical and legal issues; guidelines for oncogenetic counseling; the development of oncogenetic testing within the unique Israeli reality of the limited spectrum of *BRCA1* and *BRCA2* mutations; high-risk vs. population screening; and the definition and implementation of guidelines for surveillance of asymptomatic mutation carriers. Since 1995, oncogenetics has been transformed from a rare oddity to a pivotal player, and it represents a successful example of implementing personalized preventive medicine by identifying and providing care and by offering means for early detection and risk reduction for adults who are genetically predisposed to develop a potentially life-threatening disease—cancer in this case. Lastly, I outline my personal vision for the possible way forward for oncogenetics.

## In the beginning

“Oncogeneticist? Never heard of this. What kind of a specialty is this?”

“Why do I need to know my cancer risk? This is TMI. You are going to cause women to be depressed and even commit suicide.”

“I am an oncologist. I can provide all the information on genetics and cancer risk to all my patients. No need for oncogenetic counseling.”

This is just a short and non-representative list of some of the comments and responses that I was receiving in early 1995.

Despite the notion that cancer does cluster in families, a concept spearheaded by the late Henry Lynch in the mid-1970s (Lynch, 1974), the application of genetic testing to validate the clinical impression of an inherited predisposition to cancer was initially limited to a few high-risk, high-penetrance genes that underlie rare monogenic disorders (e.g., *Rb*, *APC*, *VHL*, and *RET*). The rapidly evolving sequencing technologies and the concomitant reduction in sequencing costs, combined with the explosion of discoveries of cancer susceptibility genes underlying more commonly encountered cancer types—breast, colon, and ovary—have led to the notion of creating a clinical platform that would address the needs of high-risk individuals—an oncogenetic service. Indeed, in the early-to-mid-1990s, the cloning of the two *BRCA* genes (Miki et al., 1994; Tavtigian et al., 1996) and the Lynch syndrome genes (Leach et al., 1993; Papadopoulos et al., 1994) led to the visionary recognition by the management of the Sheba Medical Center of the clinical need to establish an oncogenetics service. This was uncharted terrain in Israel: there was no awareness among the medical community within or outside hospitals, and there were no definite answers as to the penetrance of pathogenic variants in the genotypable genes, no established guidelines on how to define and access target populations, no well-established genetic counseling guidelines in the pretest domain or on disclosure of genetic test results, and no data on the long term psychosocial impact of being a “mutation carrier.” There were more unknowns than knowns. Here, I present a personal description of some of the insights I have had that are relevant to oncogenetics and the means used to address these issues; I provide a reflection on where we are now and offer a personal perspective for the future.

## Targeted populations—from high-risk individuals to population screens

“How do we identify eligible individuals for oncogenetic counseling?”

“Should oncogenetic counseling be limited to cancer-affected cases?”

“ Is oncogenetic counseling on a clinical basis ethical at this stage?”

Initial referrals for oncogenetic counseling underwent a rigorous selection process. Each candidate underwent a telephone interview in which he/she disclosed the relevant personal or family history of cancer, and each one was individually evaluated for eligibility by a geneticist/genetic counselor; an assessment was also made by a clinical psychologist to assess the ability of the “counseling candidate” to receive oncogenetic counseling and testing without any foreseeable deleterious psychological effects. Rigorous eligibility guidelines were designed to focus only on very high-risk cases: e.g., three cases of breast cancer in two consecutive generations, with one being diagnosed under age 50 or having bilateral breast cancer, or a cancer-patient-only counseling policy. These restrictive criteria were maintained for about 4 years and were subsequently modified based on several developments: the detection of recurring mutations in the *BRCA* genes in Ashkenazi Jews (AJ), and findings on the rate of these mutations in consecutive unselected ovarian cancer cases and the general AJ population (Modan et al., 1996; Roa et al., 1996); the identification of non-AJ recurring mutations (Theodor et al., 1998); the development of a simple, efficient technique for genetic testing for these recurring mutations as a first-pass genotyping step; and, last but not the least, an increase in awareness among physicians (especially oncologists and gynecologists) and among the public of the clinical utility of oncogenetic counseling and testing. These realities have led to relaxation of the selection criteria and streamlining of the process, enabling provision of oncogenetic counseling to all ovarian cancer patients in Israel; to all breast cancer patients who were diagnosed at under 50 years of age, especially those of AJ origin; and, in addition, to unaffected first-degree relatives of ovarian cancer patients and asymptomatic women with a significant family history. These relaxed criteria have led to an increase in the number of referrals: from less than 150 in 1995 to more than 1200 in 2004.

The next step in relaxation of the oncogenetic counseling criteria came in 2004, when the Israeli Ministry of Health (MoH) enumerated an eligibility list of individuals whose oncogenetic counseling was to be covered by the Israeli “health services basket” (Health.Gov, 2004). Soon after this, oncogenetic services were exclusively provided to this group, until the point at which the genetics institute was expanded to provide oncogenetic counseling for consecutive breast and ovarian cancer (BC/OvC) cases at the Oncology Institute Sheba Medical Center, in order to ensure a timely response in regard to the need to determine *BRCA* status as a part of the routine workup of BC/OvC cases. The institute was further augmented when it became clear that *BRCA* carriership has therapeutic implications in terms of PARP inhibitor therapy (Rouleau et al., 2010). The pivotal studies led by Prof. Ephrat Levy Lahad, showing that the penetrance of the predominant AJ *BRCA* mutations in the population is similar to that observed in high-risk families and that by applying selection criteria to the Israeli population, about half of *BRCA* mutation carriers would be missed (Gabai-Kapara et al., 2014; Levy-Lahad et al., 2014), led to a breakthrough decision in January 2020 that all cancer-free AJ women (even those with only one grandparent of AJ origin) of all ages should be offered genotyping for the three predominant mutations in the *BRCA* genes, with no need for pretest counseling, but rather taking the form of a population screen (Health.Gov, 2020). Currently, close to 75,000 AJ women have been genotyped in this context (personal communication). Given the ever-decreasing costs of genotyping, the clear clinical utility of adhering to established surveillance and early detection schemes, the availability of risk-reducing surgeries, and greater public awareness and acceptance of the possibility of cancer risk testing, I anticipate (and hope) that population-based screening for all actionable cancer-susceptibility genes will be offered soon in a cost-effective manner.

## Oncogenetic counseling and testing

“What data do you need to collect in the course of oncogenetic counseling?”

“Do we have to provide face-to-face (F2F) pretest counseling for all counselees?”

“Who should we offer F2F disclosure of test results?”

“Why can’t I test my daughter at 12 years of age without her knowledge? I want to know what her genetic status is”

In 1995, there were no readily available questionnaires in Hebrew that focused on collecting data relevant to the evaluation of the possibility of an inherited predisposition to cancer. Moreover, the algorithms that enable more structured assessment of the risks of being a *BRCA* carrier and an individual’s personal risk of developing BC/OvC (e.g., BRCAPRO) were in their infancy and not as accurate as they are now. In order to maximize our ability to collect data that seemed relevant to oncogenetic counseling, we constructed our own questionnaire based on a literature search for factors known to affect cancer risk; these included primarily family history, but also reproductive factors, personal habits, and anthropomorphic measures, to name a few. In hindsight, some of the data collected made no sense in the Israeli reality. Over the years, we have consistently asked about alcohol consumption, and of the 35,000+ women who have undergone oncogenetic counseling at the Oncogenetics unit of the Sheba Medical Center, none have reported consuming more than one or two drinks per week. Moreover, data that were not corroborated by medical documents or based on objective definitions (e.g., past surgery, precise cancer type) seemed to be skewed and inaccurate: height (for men), weight (for women), smoking status (“I marked ‘non-smoker’ because I stopped smoking last month after 40 years of smoking one pack a day”), engagement in sports (more noticeable in men), and reproductive factors (for women in the older age group). The paucity of existing, easy-to-use algorithms for visualization of the pedigree (or the prohibitively high cost of existing ones) also led to the use of hand-drawn pedigrees, with all the inherent difficulties of deciphering the shorthand and the handwriting (even by myself for my own pedigree drawings).

Six upgrades were made to the questionnaires over the years, with the current format having been stable since 2015: the questionnaires were made more streamlined by foregoing questions that were consistently answered in the negative or were non-contributory, and items collecting more relevant information were added, such as email addresses rather than landline telephone numbers. During the COVID-19 pandemic, these questionnaires have been administered online, and the possibility of online genetic counseling has been advanced, but the concept remains imperfectly developed. One of the issues that needs to be resolved is pedigree drawing: this is urgently needed, preferably at an affordable price and in a user-friendly, app-adoptable form.

## Development of guidelines and recommendations for actions to be taken by *BRCA* carriers—high-risk clinics and the “one-stop shop” concept

“So, what do I do when I leave your office, Dr Friedman?”

“Who is going to take care of me now?”

“How will I keep up with new developments relevant to my health?”

“Is it safe to take oral contraceptives and hormone replacement therapy?”

A recurring theme after a woman was informed that she was a *BRCA* mutation carrier in the late 1990s was “what do I do now?”. Indeed, the only available option in Israel at that time was to refer these women to individual specialists (a breast surgeon, a radiologist, a gynecologist) and to refer the relevant family members for oncogenetic counseling. There was no straightforward way to keep these *BRCA* mutation carriers or other affected family members updated on developments relevant to their health and surveillance guidelines, nor was there any solution in terms of a comprehensive assessment of the short- and long-term psychosocial effects of being a “high-cancer-risk individual.” It became painfully clear that there was in fact a clinical need to provide a “one-stop shop” for all high-risk *BRCA* mutation carriers. To this end, through the devoted efforts of Joel Feldshaw (who was personally affected by the loss of his (*BRCA* carrier) daughter, Meirav, to breast cancer at a young age), the Meirav high-risk clinic was established in 2007. The clinic offers a one-stop shop for carriers, providing ready access to all clinical, radiological, and (when necessary) biopsy services via the same team of healthcare professionals and secretarial support staff. Family members eligible for oncogenetic counseling are encouraged to consult with the geneticist, and informal dialog and counseling are provided by mail and/or telephone calls. The clinic also serves to keep women informed in real time of possible studies that they can join, evolving technologies (e.g., PGD), and updates to guidelines (e.g., HRT use and the timing of risk-reducing oophorectomy for *BRCA2* carriers). In my view, this is the single most important clinical innovation in oncogenetics to date.

## The future—in bullet points

• Point-of-care (POC) oncogenetic testing using POC technologies (e.g., Nanopore);
• Tele-oncogenetics for genetic counseling and disclosure of genetic test results;
• Ongoing educational efforts to enable primary care physicians to provide basic oncogenetic counseling;
• Population screening for all actionable cancer-susceptibility genes;
• One-stop shop clinics for high-risk individuals applying internationally harmonized early detection protocols and catering to the needs of all carrier family members: genetic, psychosocial, radiological, surgical, and medical;
• International collaborations and bio-genetic banks to promote research—Penetrance modifier factors; novel early detection schemes for ovarian and pancreatic cancers; definition of the missing heritability in breast cancer;
• Coverage of all procedures arising from *BRCA* mutation carrier status by the MoH/insurers.

## Data Availability

The original contributions presented in the study are included in the article/supplementary material; further inquiries can be directed to the corresponding author.
